# Pre-digest of unprotected DNA by Benzonase improves the representation of living skin bacteria and efficiently depletes host DNA

**DOI:** 10.1186/s40168-021-01067-0

**Published:** 2021-05-26

**Authors:** Yacine Amar, Ilias Lagkouvardos, Rafaela L. Silva, Oluwaseun Ayodeji Ishola, Bärbel U. Foesel, Susanne Kublik, Anne Schöler, Sebastian Niedermeier, Rachela Bleuel, Alexander Zink, Klaus Neuhaus, Michael Schloter, Tilo Biedermann, Martin Köberle

**Affiliations:** 1 Department of Dermatology and Allergology, Technical University of Munich, School of Medicine, Munich, Germany; 2 Clinical Unit Allergology Deutsches Forschungszentrum für Gesundheit und Umwelt (GmbH), Helmholtz Zentrum München, 85764 Neuherberg, Germany; 3 Institute of Marine Biology, Biotechnology and Aquaculture (IMBBC), HCMR, Heraklion, Greece; 4 Core Facility Microbiome, Technische Universität München, 85354 Freising, Germany; 5 Research Unit Comparative Microbiome Analysis, Deutsches Forschungszentrum für Gesundheit und Umwelt (GmbH), Helmholtz Zentrum München, 85764 Neuherberg, Germany; 6 DKFZ German Cancer Research Center, Berlin, Germany; 7 ZIEL - Institute for Food & Health, Technische Universität München, 85354 Freising, Germany

**Keywords:** Benzonase, DNA extraction, Next-generation sequencing, Skin microbiome, 16S rRNA, Diversity, Skin, Live/dead, Low biomass

## Abstract

**Background:**

The identification of microbiota based on next-generation sequencing (NGS) of extracted DNA has drastically improved our understanding of the role of microbial communities in health and disease. However, DNA-based microbiome analysis cannot *per se* differentiate between living and dead microorganisms. In environments such as the skin, host defense mechanisms including antimicrobial peptides and low cutaneous pH result in a high microbial turnover, likely resulting in high numbers of dead cells present and releasing substantial amounts of microbial DNA. NGS analyses may thus lead to inaccurate estimations of microbiome structures and consequently functional capacities.

**Results:**

We investigated in this study the feasibility of a Benzonase-based approach (BDA) to pre-digest unprotected DNA, i.e., of dead microbial cells, as a method to overcome these limitations, thus offering a more accurate assessment of the living microbiome. A skin mock community as well as skin microbiome samples were analyzed using 16S rRNA gene sequencing and metagenomics sequencing after DNA extraction with and without a Benzonase digest to assess bacterial diversity patterns. The BDA method resulted in less reads from dead bacteria both in the skin mock community and skin swabs spiked with either heat-inactivated bacteria or bacterial-free DNA. This approach also efficiently depleted host DNA reads in samples with high human-to-microbial DNA ratios, with no obvious impact on the microbiome profile. We further observed that low biomass samples generate an α-diversity bias when the bacterial load is lower than 10^5^ CFU and that Benzonase digest is not sufficient to overcome this bias.

**Conclusions:**

The BDA approach enables both a better assessment of the living microbiota and depletion of host DNA reads.

**Video abstract**

**Graphical abstract:**

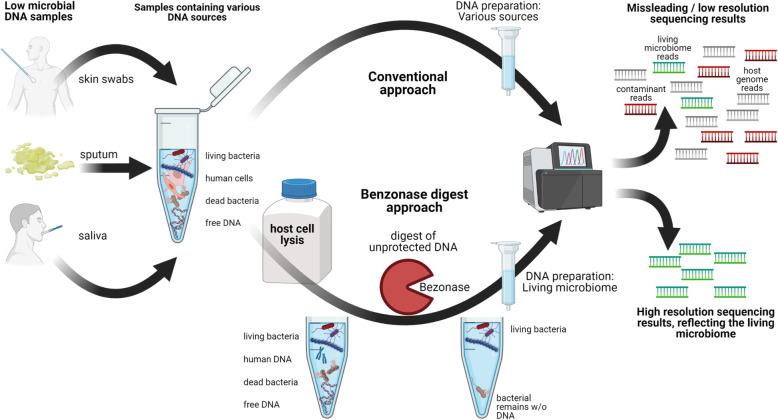

**Supplementary Information:**

The online version contains supplementary material available at 10.1186/s40168-021-01067-0.

## Background

The human skin constitutes an essential physical, chemical, and immunological barrier to the surrounding environment. It is covered by commensal microbiota, which contribute to the health of their host. In cases of dysbiosis, however, the microbiome can facilitate the colonization of the skin with facultative or obligate pathogens and, thus, initiate or exacerbate various skin diseases [1, 2]. Therefore, the analysis of skin microbiota is important for elucidating the still unclear etiologies of many skin disorders with likely microbial involvement, like atopic dermatitis [3–5], psoriasis [6, 7], or acne vulgaris [8].

The use of cultivation-independent 16S rRNA gene sequencing to analyze the composition of the skin microbiome has drastically facilitated this task and yielded a wealth of new insights, even though various concerns about validity of the output have been raised [9]. Inevitably, this approach is strongly dependent on the quality of the microbial DNA analyzed. The DNA extraction protocol therefore plays a crucial role in the final outcome. Nevertheless, most of the available protocols for bacterial DNA extraction from human specimens have been originally designed to analyze soil microbiota [10]. They are also sufficiently and widely used to prepare DNA for analyses of stool microbiomes, since both sample types contain large amounts of microbes and require removal of PCR inhibitors [11]. In contrast, microbial biomass is rather low on the skin due to cutaneous low pH and permanent secretion of antimicrobials [12]. This makes the extraction of microbial DNA from skin samples far more challenging, and optimizations of the extraction and sequencing protocols are still needed [13].

In addition to the low microbial DNA content, high ratios of human-to-microbial DNA have been reported for skin swabs [12] and various other clinical specimens, such as sputum [14], saliva [15], oral samples [16], and vaginal samples [17]. Human-to-microbe DNA ratios can even increase when samples are taken from inflamed or infected sites because of immune cell influx, tissue wounds, or necrosis [18]. Depending on the primers used to amplify the 16S rRNA gene region of choice, a strong bias may be introduced by co-amplification of non-target DNA; especially human mitochondrial 16S rRNA genes can be preferentially amplified due to their high loads [19]. Metagenome analyses of such samples are also challenging since host DNA reads can drown microbial reads, leading to a drastic increase of costs because of the higher sequencing depth required.

Several strategies have been proposed to optimize DNA preparation from samples low in microbes. For instance, methylated CpG-poor DNA is depleted to concentrate microbial DNA [20]. Other approaches employ a pre-lysis step of host cells followed by DNase digestion prior to the extraction of microbial DNA. The latter approach yielded promising results when applied on samples from resected arthroplasty components [21] or saliva [15]. Nevertheless, while these reports have proven that human DNA concentrations in microbial DNA preparations can be reduced, the elimination of DNA from dead microbiota has been neglected thus far, and the impact on community structure and functional characteristics deduced from dead microbes remains unclear. Therefore, selectively analyzing only living microorganisms might lead to a less biased interpretation of microbial communities’ composition and their active metabolic processes on the skin [22].

The aim of this study was to evaluate the ability of a DNA pre-digest approach using Benzonase to improve the representation of living skin microbiota in sequencing reads [23, 24] and to deplete host DNA. Benzonase removes host DNA and unprotected microbial DNA because of its broad activity towards DNA substrates, which it cleaves into short fragments of ≤ 5 nucleotides in length that cannot be amplified anymore. A slight preference for G/C-rich segments has been reported, but since the digest is performed before lysis of intact bacteria, no bias to the living microbiome should be introduced into analysis [23, 24]. Finally, we evaluated the impact of this approach on microbiome profiles and assessed its ability to reduce the diversity bias that might be generated in low biomass samples such as skin swabs. We demonstrated the optimized protocol to allow a more accurate interpretation of microbiome composition. This might enable a better assessment of host–bacteria interactions, since it is only the living fraction of the microbiome that can proliferate and adapt to shape a given environment.

## Results

### DNase digest prior to microbial lysis efficiently depletes unprotected DNA of dead bacteria in a skin microbiome mimicking mock community and in skin microbiome samples

The cutaneous surface provides a tough environment for its inhabiting microorganisms. Dryness, acidic pH, sparse nutrients, and antimicrobials produced by the host and competing microorganisms alike efficiently limit the quantity of bacteria living on the skin [12, 25, 26]. The resulting low bacterial DNA yield from skin samples represents a major challenge when analyzing the skin microbiome. In contrast to high microbial biomass samples (e.g., feces), any contamination from kit reagents or the laboratory environment might be prominently represented [27, 28].

We used a skin mock community to test whether DNA digest prior to bacteria lysis might improve the outcome of 16S rRNA gene sequencing by removing reads originating from dead bacteria or contaminant-free DNA. This mock community was comprised of ten species belonging to six different genera commonly isolated from human skin and representative of the three skin-dominant bacterial phyla: *Firmicutes*, *Actinobacteria*, and *Proteobacteria*. Species typically isolated from the antecubital crease (*Staphylococcus aureus, Staphylococcus epidermidis, Staphylococcus hominis,* and *Micrococcus luteus*) were included because the area is a predilection site of atopic dermatitis. This disease is an intensely studied inflammatory skin disorder with a still enigmatic role of lesional skin microbiota [29], which are not only seen as drivers of disease exacerbation but also contributors of anti-inflammatory stimuli [30–32]. Furthermore, typical skin-resident bacteria such as *Corynebacterium pseudodiphthericum, Corynebacterium striatum*, and *Bacillus horneckiae* as well as bacteria that can be found on the skin at lower abundance, like *Escherichia coli*, *Pseudomonas aeruginosa*, and *Proteus mirabilis*, were included. We added 10^7^ CFU of each bacterium to the mix to obtain final samples of 10^8^ CFU referred to as the “live” mock community. To also simulate the presence of dead bacteria, all strains were included alive except *P. aeruginosa* and *P. mirabilis*, which were added after heat inactivation (1 h at 56 °C) to the “hi” variant of the mock community. In addition, to investigate consequences of extracellular DNA, the “hi DNA” mock community consisted of the “hi” mock community, with added purified *Bacillus simplex* DNA corresponding to 10^7^ CFU (see Table 1).

**Table 1:** Bacterial species included in the skin microbiome mimicking mock community used for BDA and NDA comparisons. *Pseudomonas aeruginosa* and *Proteus mirabilis* were added heat-killed to the hi mock community. The hi DNA mock community additionally contained purified *B. simplex* DNA

Gram positive	Gram negative
*Staphylococcus epidermidis* (*Firmicutes*)	*Escherichia coli* (*Proteobacteria*)
*Staphylococcus hominis* (*Firmicutes*)	*Pseudomonas aeruginosa* (*Proteobacteria*)
*Staphylococcus aureus* (*Firmicutes*)	*Proteus mirabilis (Proteobacteria)*
*Micrococcus luteus* (*Firmicutes*)	
*Corynebacterium pseudodiphthericum* (*Actinobacteria*)	
*Corynebacterium striatum* (*Actinobacteria*)	
*Bacillus horneckiae* (*Firmicutes*)	

**Fig. 1:**
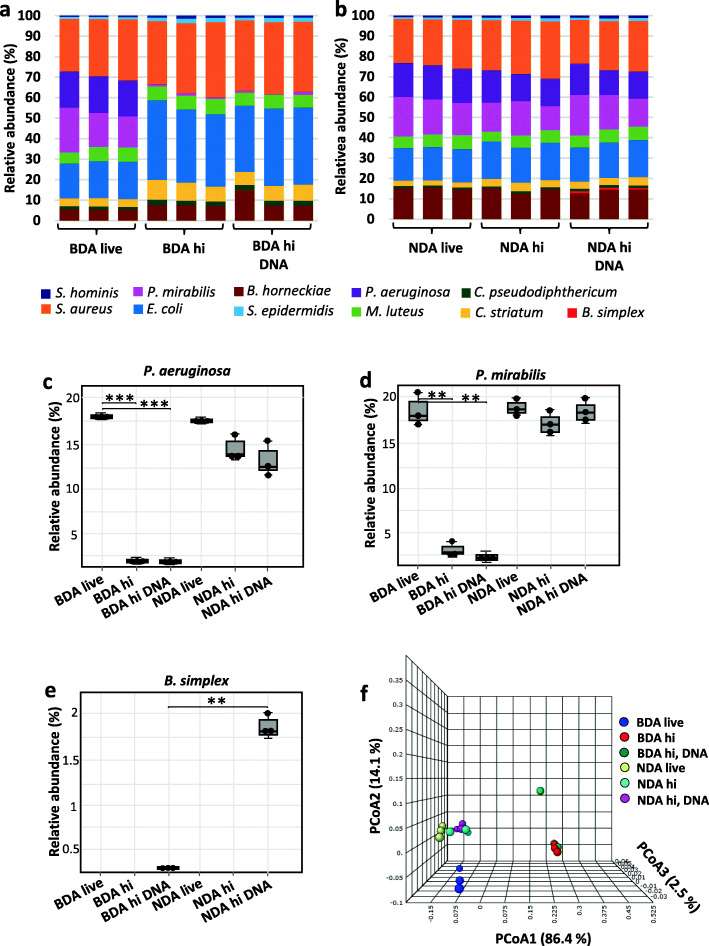
Benzonase digest efficiently depletes DNA from dead bacteria and free bacterial DNA in skin mock community samples. Microbial DNA was extracted using **a** a Benzonase-digest approach (BDA) or **b** without Benzonase pre-digest (NDA). Relative OTU abundance of reads obtained from amplified 16S rRNA genes are shown. The mock community consists of living bacteria (live, left), including heat-inactivated (hi, middle) bacteria (*P. aeruginosa* and *P. mirabilis*) and additional free *B. simplex* DNA (hi DNA, right). **c** Relative abundances of *P. aeruginosa* and **d** *P. mirabilis* subjected (hi) or not (live) to heat inactivation before DNA extraction based on 16S rRNA gene sequencing of mock communities. *p* values were calculated using Wilcoxon-Mann-Whitney test. * *p* ≤ 0.05, ** *p* ≤ 0.01, *** *p* ≤ 0.001. **e** As panel c, but showing free DNA of *B. simplex*, spiked in before DNA extraction. **f** Principal coordinate analysis (PCoA) plot of β-diversities for different skin mock communities extracted using BDA or NDA

We then compared 16S rRNA gene sequencing data after DNA extraction using the QIAamp DNA Microbiome kit, preceded (BDA) or not (NDA) by a Benzonase digest that removed all DNA not protected by an intact bacterial cell envelope. Although bacteria have been added at similar initial loads, OTU’s relative abundances obtained from amplified 16S RNA genes differed considerably (BDA / NDA live; Fig. 1a and b, left). This is likely due to differences in 16S rRNA gene copy numbers between bacterial species as well as varying susceptibilities to mechanical and enzymatic lysis during bacterial DNA extraction. When *P. aeruginosa* and *P. mirabilis* cells were heat inactivated, reads from these bacteria sharply declined in BDA-processed samples and their relative abundances dropped from 17.75 ± 0.13% (*P. aeruginosa*) and 17.83 ± 2.91% (*P. mirabilis*) to 0.041 ± 0.03% and 0.99 ± 0.09%, respectively (hi; Fig. 1a, middle; Fig. 1c and d). Application of the BDA to the hi DNA mock community resulted in virtually complete degradation of the extracellular DNA (0.001% remaining reads from *B. simplex*). In contrast, the extracellular DNA in the hi DNA mock community was mostly unaffected by the NDA (Fig. 1a, right; Fig. 1b, right; Fig. 1e). This demonstrates that DNA originating either from dead bacteria or free bacterial DNA was efficiently removed, which otherwise would result in similar operational taxonomic unit (OTU) read numbers (i.e., a similar microbiome composition) as seen with NDA samples prepared without pre-digest (Fig. 1b–e). Interestingly, depletion of dead bacteria and free bacterial DNA led to distinct clustering in a principal coordinate analysis (*PCoA*; Fig. 1f), demonstrating that the presence of large amounts of dead bacteria within microbiome samples introduced a bias into data interpretations in terms of structure and functionality.

We verified these results by comparing BDA with a different and more conventional microbiome DNA preparation approach using the ZymoBiomics DNA miniprep kit without any pre-digest (CA). To this end, we analyzed a smaller skin mock community (Fig. S1a) and a microbiome suspension prepared from a human antecubital crease swab (Fig. S1c). These preparations were spiked either with 10^6^ heat-inactivated *E. coli* cells or 4.2 ng of purified *E. coli* DNA. As expected, OTU reads matching to *E. coli* dominated the 16S rRNA gene analysis of CA-prepared samples in both cases. In contrast, BDA led to strong reduction of reads from either dead *E. coli* cells or its free DNA (Fig. S1b, d).

Taken together, BDA improves the representation of bacterial taxa truly present and alive in skin swabs and other low microbial density samples. Noteworthy, the additional preparation steps introduced to perform Benzonase digest in BDA did not result in any other obvious microbial DNA contamination.

### Human DNA is efficiently depleted by the BDA approach with no impact on the microbiome profile

**Fig. 2:**
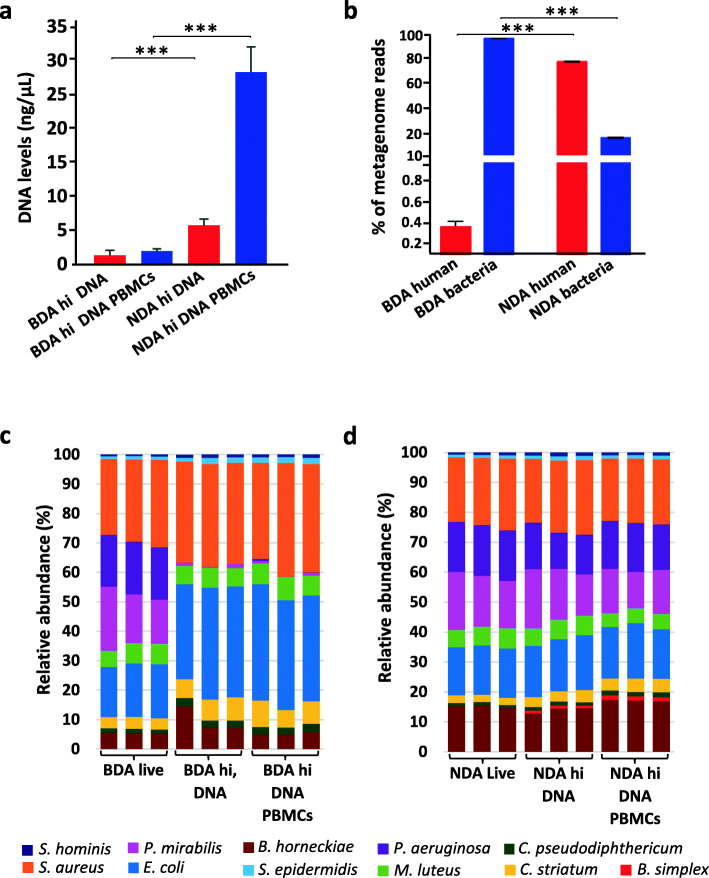
Human DNA is efficiently depleted by the BDA approach with no impact on the microbiome profile. **a** DNA yields from skin mock community samples with heat-inactivated bacteria and added free DNA (hi, DNA), further supplemented with 10^5^ PBMCs and processed either with BDA or NDA. *p* values were calculated using Wilcoxon-Mann-Whitney test *** *p* ≤ 0.001. **b** Percentage of reads related to humans or bacteria based on metagenomic sequencing. *** *p* ≤ 0.001. **c** Relative OTU abundance obtained from amplified 16S rRNA genes from mock community members following BDA or **d** NDA approaches

Next, we evaluated the effectiveness of BDA to remove host DNA before preparing microbial DNA. Total DNA yields from mock community samples with heat-inactivated *P. aeruginosa* and *P. mirabilis* bacteria, supplemented with purified *B. subtilis* DNA and human 10^5^ PBMCs, decreased from 28.33 ± 3.68 ng/μl in samples processed with NDA to 1.95 ± 0.3 ng/μl with BDA (Fig. 2a). Strikingly, human DNA reads found in whole metagenome analysis were significantly reduced by BDA to 0.37 ± 0.08% from formerly 80.88 ± 0.51% with NDA (Fig. 2b), indicating an efficient removal of host DNA from microbiome samples following host cell lysis and Benzonase digest. Confirming this result, BDA also diminished DNA yields compared with CA. The latter retained roughly the same amount of host DNA compared with an approach designed for DNA preparation from human blood cells (HA; Fig. S2a, b).

Importantly, the taxonomic binning at OTU level of 16S rRNA gene sequencing data did not reveal any notable changes in analyzed skin mock communities (containing heat-inactive bacteria and free bacterial DNA) supplemented or not with PBMCs and processed with BDA (Fig. 2c, middle and right). Similarly to 16S rRNA gene based sequencing (Fig. 2c and d), the community profiling using the metagenomics approach had also shown a drastic decrease of dead bacteria and complete depletion of free bacterial DNA following BDA (Fig. S3c, d). In addition, the PCoA plot of β-diversity showed strong shifts in microbiome structure when adding heat-inactivated bacteria and free bacterial DNA, but no effect exerted by the supplementation of PBMCs was observed (Fig. S2c). We conclude that the Benzonase digest step following host cell lysis in the BDA efficiently depletes host DNA as well as unprotected bacterial DNA with no significant impact on the viable microbiota fraction. Therefore, although the relative abundance of reads resulting from intact bacteria is not affected, information will still be lost if reads are wasted on human DNA.

### Low biomass microbiome samples result in a biased α-diversity estimation

**Fig. 3:**
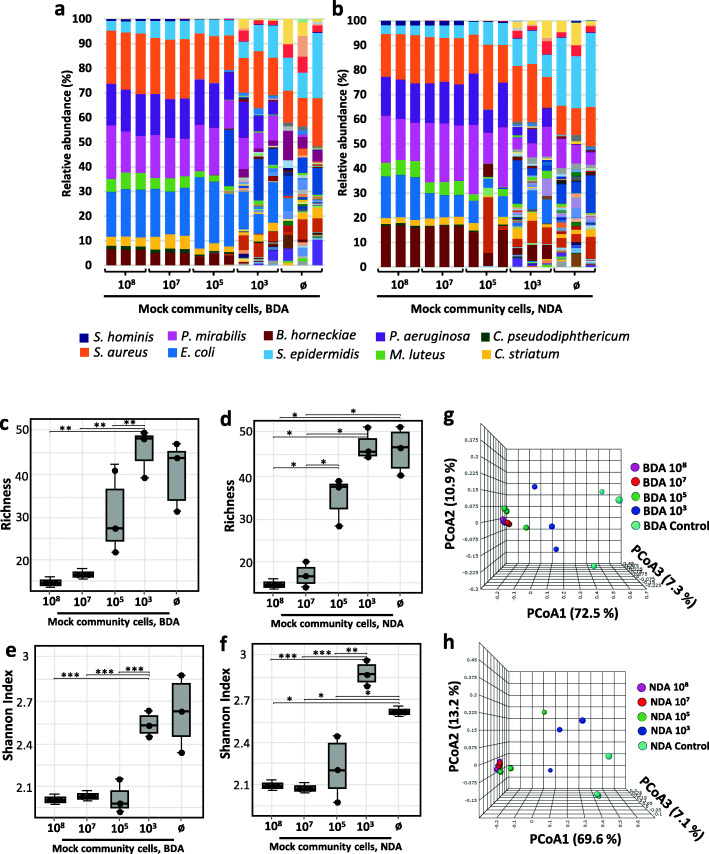
Serial dilutions of skin mock community samples display increased α-diversity. The original mock community (10^8^ CFU) was diluted to 10^7^, 10^5^, and 10^3^ CFU/sample. Relative OTU abundance obtained from amplified 16S rRNA genes upon serial dilutions followed by DNA extraction using **a** BDA or **b** NDA. The α-diversity increased upon dilution when expressed as either richness for **c** BDA and **d** NDA or Shannon diversity index for **e** BDA and **f** NDA. *p* values were calculated using Wilcoxon-Mann-Whitney test. * *p* ≤ 0.05, ** *p* ≤ 0.01, *** *p* ≤ 0.001. PCoA plots show shifting of β-diversity of diluted samples prepared by **g** BDA or **h** NDA

The low bacterial DNA content of low microbiome input samples such as skin swabs poses the danger of artificially increased diversity, i.e., the appearance of OTUs in sequencing results that do not originate from sampled microbiomes can be a crucial confounder [33–35]. To assess any introduced diversity bias, we analyzed 16S rRNA gene reads in dilution series of the skin mock community. For both BDA and NDA, we observed an increase of microbial α-diversity upon sample dilution. In 1:1000 diluted samples (i.e., 10^5^ CFU), contaminations still had a minor effect when prepared by BDA, only becoming significant for samples at or below 10^3^ CFU. At this point, numerous bacterial OTUs appeared that had not been included into the original mock community (Fig. 3a and b). In line with this finding, sequencing the sterile water control samples showed the highest number of OTUs. An even stronger increase in bacterial richness (i.e., number of OTUs) was observed with NDA, reaching a significant difference at 10^5^ CFU (Fig. 3c and d). Similarly, the Shannon index increased in both approaches, with significant differences achieved at the ultimate dilution (10^3^ CFU) (Fig. 3e and f).

PCoA showed that β-diversity of diluted samples shifted upon dilution, with PCoA1 accounting for about 70% of differences in both approaches. The microbiome composition was independent from the influence of dilution or contaminants when the bacterial load was equal to or higher than 10^5^ CFU (Fig. 3g and h). However, in comparison to samples processed by the CA, BDA showed a higher stability in terms of α- and β-diversity upon serial dilutions (Fig. S4). In samples processed by the CA, α-diversity was generally higher than in BDA samples, e.g., in the 10^5^ CFU samples richness was more than twice as high (Fig. S4c, d). This difference resulted in a distinct microbial profile which clustered remote from the BDA-processed mock community samples in multidimensional scaling (MDS) for the β-diversity, similar to the 100 times more diluted 10^3^ CFU sample after BDA preparation (Fig. S4g, h). The BDA, therefore, resulted in α-diversity values closer to the original bacterial community, especially when samples contained numbers of bacteria typically obtained from skin swabs (in our experience ≤ 10^5^ CFU/sample if sampled skin area is not strongly infected).

**Fig. 4:**
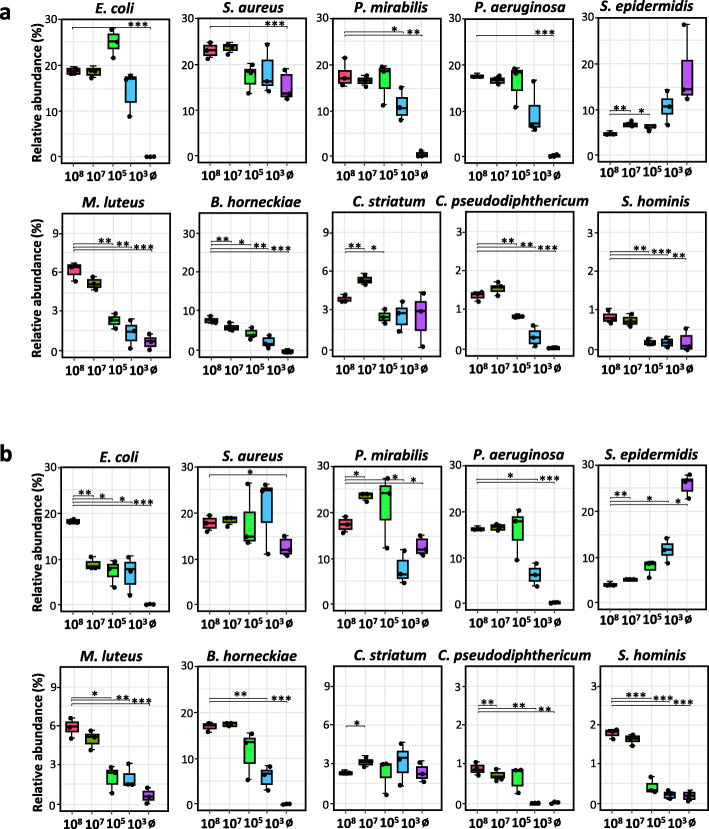
Changes of OTU abundance in mock community samples upon serial dilutions. Relative OTU abundance obtained from amplified 16S rRNA gene sequencing from mock communities processed by **a** BDA or **b** after NDA extraction decreased for most mock community strains but increased for *S. epidermidis*. *p* values were calculated using Wilcoxon-Mann-Whitney test. * *p* ≤ 0.05, ** *p* ≤ 0.01, *** *p* ≤ 0.001

We additionally performed a relative abundance analysis for each species present in the diluted mock community, processed with either the BDA (Fig. 4a) or NDA (Fig. 4b). Results showed stable or decreased relative abundance values of nearly all bacterial members of the mock community upon dilution irrespective of the approach used. However, *S. epidermidis* reads, which were highly abundant in the control samples (up to 20% in BDA and 30% in NDA), increased upon dilution. *S. aureus* was also present at a high relative abundance of about 12.5% in both controls, while *C. striatum* was detected at lower abundance (3%). Interestingly, *P. mirabilis* was exclusively present in the NDA control samples, indicating that it was introduced before Benzonase pre-digest. Additionally, spurious reads of OTUs not present at all in the original mock community made up 36.53% and 40.20% of all reads in the BDA and NDA-processed samples with 10^3^ CFU, respectively (Tables S1 and S2). Most of the contaminants detected at this dilution were either typical environmental bacteria like *Arenibacter nanhaiticus* or *Undibacterium oligocarboniphilum,* which has been isolated from purified water [36]. In lower proportions, we also detected reads belonging to the genera *Propionibacterium*, *Corynebacterium*, *Streptococcus*, and *Lactobacillus*.

However, in either approach, the number of contaminating reads was lower compared with that in the CA. This comparison between kits has been added to assess the effect of factors other than Benzonase pre-digest, e.g., of kit purity when performing microbial DNA extraction from low biomass samples. In the CA, up to 80% reads belonged to contaminants in the 10^3^ CFU dilution of the mock community (Tables S3 and S4). This is in line with studies reporting that kit reagents are a potential source of bacterial DNA contamination, which is especially relevant in low biomass or diluted microbiome samples [35, 37–39]. Most of the contaminating bacterial OTUs found are from species ubiquitously present in soil and water. They are apparently able to contaminate the DNA extraction kits during production at different abundances depending on the manufacturing process. In contrast, contaminations introduced by sampling, the PCR or the performing lab seem to play a rather minor role compared with kit contaminants, as only 7 of 34 contaminating OTUs appeared in any BDA sample and 7 of 38 in any CA sample. These contaminations seem to be very low, since not all OTUs found in the kit controls were also observed in samples using 10^3^ CFU.

## Discussion

The human microbiome plays a central role in host health and disease. Dysbiotic shifts of its composition have been linked to the genesis and progression of a wide variety of diseases, such as atopic dermatitis, psoriasis, asthma, colitis, and obesity [6, 40–43]. Various techniques have been developed for characterizing the microbiome, but approaches that employ NGS, like 16S rRNA gene sequencing or metagenome analyses, have gained increasing interest by achieving this goal at ever lower costs and unchallenged accuracy so far [39, 44]. However, most protocols for DNA isolation do not discriminate between DNA of viable and dead bacteria, thus introducing a bias into NGS-based analysis results [45]. This limitation significantly affects the data and may lead to misinterpretations, since the structure and functions of microbiomes are strongly linked to the vitality of their community members [46].

### Removal of DNA from dead bacteria

We investigated the feasibility of a Benzonase-based approach to pre-digest unprotected DNA to overcome these limitations and thus offer a better assessment of the living microbiome. On the downside, this approach adds almost 2 hours of incubation and hands-on time in addition to requiring freshly prepared samples to avoid bacterial lysis by freezing. We focused on skin samples because their microbiome is challenging to analyze due to the low numbers of bacteria present [47, 48]. BDA not only completely removed purified *B. simplex* DNA but also drastically reduced reads in the final data originating from heat inactivated bacterial cells spiked into the mock community (*P. aeruginosa*, *P. mirabilis*) and skin samples (*E. coli*). Originally designed to remove unwanted human reads in metagenome sequencing by host cell pre-lysis followed by DNA digest, BDA therefore noticeably decreases reads from dead bacterial cells and free DNA. This improves the characterization of the living microbiome by both 16S rRNA gene and metagenomics sequencing.

One of the major advantages of assessing the living microbiome is that it enables a more accurate interpretation of host–bacteria interactions. The structure and function of the microbiomes is mainly dependent on members of the community that are alive. Certainly, microbes without intact cell walls can be considered dead, but they can constitute a large fraction of the OTU reads in sequencing based approaches [49]. Occasionally, bacteria enter a state called viable but not culturable (VBNC) [50]. Such cells may not be active members of the microbiome at the time of sampling and will not be detected by culture-based methods, but they may still be important in earlier or later stages of a disease. Therefore, it would be often desirable to link the detected microbial species with quantitative data of their metabolic activity, since we expect BDA not to digest DNA of still intact VBNC cells. Methods for the detection of metabolic activities cannot be easily applied to low biomass samples, however, and do not yet enable reliable assessment of metabolic activity [46].

### Host DNA removal

An additional advantage of BDA is removal of host DNA reads. It has been reported that metagenomes of human stool samples contain usually less than 10% of reads mapping to the host’s genome, while skin, nasal cavity, vaginal, and sputum samples yield up to 90% of human reads [15]. Remarkably, BDA works without affecting the microbiome profile in terms of α- or β-diversities. Indeed, our metagenomics data analysis revealed a drastic and significant decrease of host DNA reads from 81 to 0.37% when using BDA, thereby increasing bacterial reads in these samples with high human DNA load to up to 99.63%. Corroborating our results, Nelson et al. [18] have shown a noticeable decrease of human and extracellular DNA reads following a Benzonase digest in sputum samples, which dropped from 96% with the standard extraction method to 60%, along with an up to 15-fold increase of bacterial reads.

Efficient depletion of host DNA is of great importance in metagenomics analysis, since host DNA generates immense amounts of unwanted reads. Some protocols rely on the viability PCR method, using ethidium monoazide (EMA) or propidium monoazide (PMA) staining to mitigate this problem [15, 46, 51]. These dyes penetrate damaged cells to intercalate with their DNA. Exposed to blue light, the dyes form covalent bonds, preventing DNA amplification [52–54]. However, these dyes have been shown to penetrate various intact bacteria, including *E. coli*, *Staphylococcus aureus*, *Streptococcus sobrinus,* and *Mycobacterium avium* [55], and display antimicrobial effects as observed for *Listeria monocytogenes* and *Legionella pneumophila* [56, 57]. Finally, penetration of living bacterial cells can vary depending on their physiological state (e.g., the uptake is increased in rapidly dividing cells compared to senescent cells) [58]. Furthermore, Nelson et al. [18] argued that the efficiency of PMA is limited to the amplification of long targets (amplicon sequencing), while metagenomics sequencing may yield smaller fragments, which are less affected. In another study, however, combining host cell lysis with PMA treatment to process human saliva samples, Marotz et al. have shown an effective removal of host-derived sequencing reads from about 90% in untreated samples down to about 9% after treatment [15]. Thus, PMA might be useful under some circumstances, while BDA seems to be more effective and more robust for different sample and analysis types.

### Contaminating DNA

Concerning microbial diversity, we demonstrated that contaminating bacterial DNA might considerably affect microbiome profiles. We have observed increasing values of α-diversity in samples prepared by both BDA and NDA upon serial dilutions. Increased diversity was more obvious in low bacterial input samples containing 10^3^ CFU for both BDA and NDA, while CA showed large numbers of non-sample OTUs already when the number of bacteria in the sample was two orders of magnitude higher (10^5^ CFU). These results were concordant with the β-diversity data plots, which display stronger shifts of the diluted CA samples compared to the diluted BDA-samples towards the control’s position. Two principal sources of contamination have been reported in microbiome studies: contaminant DNA and cross-contamination [39]. Contaminating DNA can originate from various sources, including sampling procedures and environments [38, 59, 60], DNA extraction kits [34, 35], and laboratory reagents like PCR master-mixes [61]. Cross-contamination occurs from other adjacent samples, sequencing runs, and barcode leakage [62]. Our data indicate that kits or reagents play an important role in the contamination of low biomass samples processed here. This contamination distorts microbiome analysis. The BDA did not reduce the appearing α-diversity bias for highly diluted samples, but lower contamination introduced by reagents and consumables used in both BDA and NDA resulted in much better results compared with the CA approach. Thus, a careful analysis and interpretation of microbial diversity is needed, especially when working with low biomass samples such as skin, lung, or blood samples [63].

For instance, the α-diversity interpretations in chronic skin pathologies, like atopic dermatitis, should be reconsidered, since the low diversity observed in lesional skin samples could be due to a high number of reads from one bacterial species (*S. aureus* in this case), which dilutes out any other OTUs still present, albeit at much lower abundances. On the other hand, based on the noted increase of diversity upon serial dilutions, one can imagine that the high α-diversity observed on the skin of healthy controls in many microbiome studies might at least partially be due to the low number of bacteria captured from the hostile cutaneous environment. To ensure highly reliable results from samples with very low bacteria content, collecting the maximum sample amount is critical. Furthermore, it might be feasible and helpful in some studies to quantify the bacterial number in samples by counting bacteria cultured from a fraction of the sample or by qPCR quantification to exclude samples with too low bacterial content (i.e., ≤ 10^3^ CFU/sample).

## Conclusions

In this study, we examined the ability of a Benzonase-based approach for microbial DNA extractions to improve the representation of living microorganisms in human skin microbiome samples. This method successfully decreased read numbers from dead bacteria or extracellular DNA. The BDA approach efficiently depleted host DNA reads with no significant impact on the microbial profile in metagenomes. Moreover, we have shown that more diluted samples from mock communities (i.e., with very low amounts of bacterial DNA) display an increased α-diversity due to spurious OTUs. This highlights the need for careful interpretations of data from low biomass samples, e.g., from the skin of healthy controls with effective host defenses, where reagent and other contaminants may play a major role.

## Methods

### Skin mock community

Representative bacteria of six different genera have been isolated from the antecubital crease of healthy and atopic participants in an adult atopic eczema cohort at the dermatology hospital of the Technical University Munich. Isolated bacterial strains were purified by subculturing on TSA media. Stock suspensions were stored at – 80 °C after adding glycerol (10%). Bacteria were identified by microscopy, biochemical analysis using api® Staph / 20 NE strips (bioMérieux, Nürtingen, Germany), and MALDI-TOF.

To compare bacterial diversity between BDA and NDA-purified DNA, a skin mock community of ten strains was used (Table 1). The mock community was assembled by mixing concentrations of 10^7^ CFU per strain each to establish a final mock community of 10^8^ CFU (“live” mock community). To evaluate the ability of BDA to eliminate DNA from dead bacteria, *P. aeruginosa* and *P. mirabilis* were not added to the mix vital as in the live mock community but after a heat inactivation step of 1 h at 56 °C (“hi” mock community). Further, purified *B. simplex* DNA corresponding to 10^7^ CFU was added to the hi mock community to assess the effectiveness of BDA in depleting free bacterial DNA reads (“hi DNA” mock community). Finally, we evaluated whether the BDA was able to remove host DNA reads from skin mock community samples by adding 10^5^ peripheral blood mononuclear cells (PBMCs) to the hi DNA mock community.

To compare BDA with a more conventional DNA extraction approach (CA), four bacteria, namely *Staphylococcus aureus*, *Micrococcus luteus*, *Corynebacterium pseudodiphtericum*, and *Moraxella osloensis* were included in the skin mock community. The mock community was created by adding 2 × 10^7^ CFU of each bacterium. Next, 2 × 10^7^ CFU *E. coli*, either living or heat inactivated (56 °C for 1 h), or 58 ng of purified DNA (corresponding to 2 × 10^7^ CFU) were spiked-in. In total, an undiluted mock community sample consisted of 1 × 10^8^ CFU re-suspended in 1 ml 0.15 mM NaCl solution.

To examine low-DNA input samples, bacterial suspensions were prepared from 10^8^ CFU, which were then diluted to 10^7^, 10^5^, or 10^3^ CFU, respectively. Nuclease free water was used as control.

### Human skin microbiome samples

Skin swabs were obtained from the arm fossa of a healthy volunteer by rubbing back and forth approximately 50 times and applying firm pressure using forensic 4N6FLOQ swabs (COPAN flock technologies, Brescia, Italy) moistened with 0.15 mM NaCl solution containing Tween 20 at 0.1% [64, 65]. Microbiota were released from swabs by swirling in 15 ml of NaCl solution at 0.15 mM. Skin microbiome samples were supplemented with heat-inactivated *E. coli* cells (1.4 × 10^6^ CFU/sample) or its purified DNA (4.2 ng/sample corresponding to 1.4 × 10^6^ CFU).

### DNA extraction

Microbial DNA was extracted from mock communities or skin microbiome swabs using a commercial microbiome DNA extraction kit and either including (BDA) or not including (NDA) a 30-min Benzonase pre-digest of unprotected DNA according to the manufacturer’s instructions (QIAamp DNA Microbiome kit; Qiagen, Hilden, Germany). Samples were extracted freshly to avoid bacterial lysis by freeze-thawing. An additional microbial DNA extraction method (CA), not designed to include a Benzonase digest (ZymoBIOMICS DNA miniprep kit; Zymo Research, Freiburg, Germany), was used for comparison (following the manufacturers’ protocol). Both kits use a combination of mechanical and chemical lysis to disrupt Gram-negative and Gram-positive bacteria. Bacterial DNA is purified through adsorption to silica membrane columns included in the kits, which have undergone a proprietary DNA decontamination process. The Maxwell® 16 LEV Blood DNA Kit (Promega, Fitchburg, WI) was used as a control method for host DNA preparation (human DNA preparation approach; HA). All DNA samples were suspended in 50 μl elution buffer. DNA concentrations were estimated using the Quantus fluorometer (Promega); human DNA was also quantified by real-time PCR using the human gDNA detection kit (Primer Design, Camberley, UK). DNA samples were stored at – 20° C until further processing.

### 16S rRNA gene amplification

The 16S rRNA gene-specific primers used for targeting the V3 and V4 regions were, forward: S-D-Bact-0341-b-S-17 (5’ → 3’) TCG TCG GCA GCG TCA GAT GTG TAT AAG AGA CAG CCT ACG GGN GGC WGC AG, and reverse: S-D-Bact-0785-a-A-21 (5’ → 3’) GTC TCG TGG GCT CGG AGA TGT GTA TAA GAG ACA GGA CTA CHV GGG TAT CTA ATC C [66]. To each 5 μl of extracted template DNA, 12.5 μL of a NEB Next High Fidelity Master Mix (New England Biolabs, Ipswich, MA), 0.5 μL of each forward and reverse primers (10 pmol/μl), and 6.5 μl of DEPC water were added. A first PCR was performed in two replicates for every sample as follows: 5 min at 98 °C, followed by 25 amplification cycles of 10 s at 98 °C, 30 s at 60 °C, 30 s at 72 °C, and finally 5 min at 72 °C. A second PCR was performed for dual indexing with Illumina adaptors from the Nextera XT Index Kit v2 Set B (Illumina, San Diego, CA). One microliter of purified amplicon sample (10 ng) was mixed with 2.5 μl of each Illumina index, 12.5 μl of the Next High Fidelity Master Mix, and 6.5 μl of DEPC water. PCR settings were 30 s at 98 °C, followed by 8 amplification cycles of 10 s at 98 °C, 30 s at 55 °C, 30 s at 72 °C, and a final heating step at 72 °C for 5 min [66]. Indexed PCR products were purified using Ampure XP beads (Beckman Coulter, California, USA) and analyzed using Agilent DNA 7500 Chip (Agilent, Waldbronn, Germany). The DNA concentration was measured with QuantiFluor dsDNA System using a Quantus fluorometer (Promega). The sequencing library was prepared by pooling 4 nM of each purified sample equimolarly for sequencing on an Illumina MiSeq platform with a PE300 v3 cartridge (generating up to 25 million of 2 × 300 bp reads). The control samples did not generate measurable amounts of PCR amplicons and were, therefore, added to the pool using an equal volume instead. Final pools were spiked with 10% phiX.

### Metagenomic library preparation

Metagenomic libraries were constructed using the NEBNext® Ultra™ II FS DNA Library Prep Kit for Illumina® (New England BioLabs). Dual indexing was conducted employing the NEBNext® Multiplex Oligos kit for Illumina® (Dual index primers set 1, New England BioLabs). Purification and size selection was performed based on Agencourt® AMPure® XP (Beckman-Coulter, MA, USA). Library inserts ranging from 250 to 400 bp were further evaluated using a Fragment Analyzer™ (Advanced Analytical, Ankeny, IA). Libraries were pooled equimolarly and 15 pM was spiked with 1% PhiX (Illumina). Sequencing was performed on an Illumina® NextSeq 550 (Illumina) using the paired-end mode (2 × 150 bp Mid output kit bp, Kit v2.5, 300 cycles).

### Sequence analysis and statistics

Raw 16S rRNA gene amplicon sequencing reads were processed following the UPARSE method [67] as implemented in the online IMNGS platform [68]. In brief, primer and barcode sequences were trimmed from each read, and sequences shorter than 200 bp were discarded. Clusters were formed de-novo at 97% similarity cutoff and the resulting OTUs were taxonomically classified by the RDB classifier [69]. Downstream analysis of the OTU tables was performed using the R scripts available in the Rhea pipeline [70]. Experiments were performed in triplicates. Group comparisons were made using Wilcoxon-Mann-Whitney test followed by the Benjamini-Hochberg post-hoc procedure for multiple comparisons.

For the metagenomics experiment, adapters and primers were removed from raw reads using Adapterremoval v2.1 [71]. Reads with nucleotides with quality values less than 15 were trimmed at this position and sequences shorter than 50 bp discarded. Human DNA sequence reads were identified and removed using KneadData v0.35 (https://huttenhower.sph.harvard.edu/kneaddata/) with the Hg-19 human reference genome, and percentages were calculated. Taxonomy was obtained using MetaPhlAn v3.0, which uses a database of clade-specific markers to quantify bacteria constituents at the species and higher taxonomic levels. MetaPhlAn v3.0 was run using default settings [72].

Tables displaying read counts from 16S rRNA gene sequencing and metagenomics analyses, as well as initial DNA concentrations, have been included as Supplementary Data.

## Supplementary Information

**Additional file 1: Supplementary Figure S1.** Benzonase digest approach efficiently depletes dead bacteria and free bacterial DNA reads. Microbial DNA was extracted using a conventional approach (CA) or the benzonase-digest approach (BDA) before bacterial lysis. a) Microbiome analysis of a skin mock community supplemented with live *E. coli* cells, heat inactivated (hi) *E. coli* cells or *E. coli* free DNA. b) Relative abundance reads resulting from *E. coli* in the mock community. c) Microbiome analysis at genus level of forearm skin samples spiked with *hi E. coli* or *E. coli* free DNA. d) Relative abundance of *E. coli* reads in spiked skin samples. P valued were calculated using Wilcoxon-Mann-Whitney test. * *p* ≤ 0.05, ** *p* ≤ 0.01, *** *p*≤ 0.001.

**Additional file 2: Supplementary Figure S2.** Benzonase digest approach efficiently depletes dead bacteria and host DNA reads with no impact on microbiota composition. a) DNA yields from a skin mock community supplemented with 3×10^5^ PBMCs/sample and processed with different extraction methods: HA (Human blood kit), CA (conventional approach for bacteria DNA extraction) and the BDA (benzonase digest approach). b) Real-time PCR plot of human DNA reads corresponding to the mock community supplemented with PBMCs extracted using different approaches. c) The principal coordinate analysis plot (PCoA) of β-diversity shows the ability of BDA to eliminate human DNA reads from PBMCs as well as DNA from heat inactivated bacterial cells (from *P. aeruginosa* and *P. mirabilis*) and free bacterial DNA (*B. simplex*). BDA (benzonase-digest approach), NDA (non-benzonase-digest approach). PBMCs (human peripheral blood mono nuclear cells). Live (mock community comprising living bacteria), hi (heat inactivated *P. aeruginosa* and *P. mirabilis*), DNA (free DNA from *B. simplex*).

**Additional file 3: Supplementary Figure S3.** Benzonase digest approach efficiently depletes dead bacteria and host DNA from metagenomics reads. a) Metagenomics read counts from skin mock community samples (hi, DNA), supplemented or not with 10^5^ PBMCs and processed either with BDA or NDA. b) Human and bacteria metagenomics read counts in mock community samples (hi, DNA). c) Relative abundance of OTUs detected in metagenomics reads. d) Relative abundance of OTUs obtained from skin mock community supplemented or not with human PBMCs. The taxonomy analysis based on metagenomics data has been performed using MetaPhlAn 3.0 tool. BDA (Benzonase-digest approach), NDA (Non-Benzonase-digest approach). PBMCs (human peripheral blood mono nuclear cells). OTUs (Operational taxonomic units), hi, DNA (Skin mock community with heat inactivated *P. aeruginosa* and *P. mirabilis* and free bacterial DNA from *B. simplex*).

**Additional file 4: Supplementary Figure S4.** The Benzonase approach generates less diversity bias compared to CA in low bacterial DNA input samples. The mock community of living bacteria (10^8^ CFU/sample) was diluted to 10^7^, 10^5^ and 10^3^ CFU/sample and then DNA extracted by the CA or the BDA. Taxonomic binning of bacterial taxa in samples processed by a) BDA or b) CA. c, d) Richness e, f) Shannon effective and g, h) β-diversity analysis of the mock community after dilution and extraction using BDA or CA. BDA (Benzonase digest approach), CA (conventional approach). * *p* ≤ 0.05, ** *p* ≤ 0.01, *** *p* ≤ 0.001.

**Additional file 5: Supplementary table 01.** Abundant OTUs in the Benzonase digest approach (BDA) upon dilution (OTUs used to constitute the mock community in bold letters). **Supplementary table 02.** Abundant OTUs in the Non-Benzonase digest approach (NDA) upon dilution (OTUs used to constitute the mock community in bold letters). **Supplementary table 03.** Abundant OTUs (> 1%) in the benzonase digest approach (BDA) upon dilution (small mock community, OTUs used to constitute the mock community in bold letters). **Supplementary table 04.** Abundant OTUs (> 1%) in the conventional approach (CA) upon dilution (small mock community, OTUs used to constitute the mock community in bold letters).

**Additional file 6: Table 01.** 16S rRNA gene amplicon reads per sample after every processing step. **Table 02.** Metagenomics reads per sample after every processing step. **Table 03.** Metagenomics based taxa relative abundances. **Table 04.** Metagenomics based taxa relative abundances in sampled containing PBMCs.

## Data Availability

OTU tables and metagenome read counts are available as supplementary files.

## Abbreviations

BDA: Benzonase digest approach
CA: Conventional approach of microbial DNA preparation
CFU: Colony-forming unit
DEPC water: Diethyl pyrocarbonate treated water
EMA: Ethidium monoazide
HA: Human DNA preparation approach
hi: Heat inactivated
NDA: Non-Benzonase digest approach
NGS: Next-generation sequencing
OTU: Operational taxonomic unit
PBMCs: Peripheral blood mononuclear cells
PMA: Propidium monoazide
RDP: Ribosomal Database Project
rrnDB: Ribosomal RNA Database of Michigan University
TSA: Trypticase soy agar
TSB: Trypticase soy broth
