# Supplementary material for: Pre-digest of unprotected DNA by Benzonase improves the representation of living skin bacteria and efficiently depletes host DNA
Source: Microbiome. 2021 May 26;9:123. doi: 10.1186/s40168-021-01067-0 (PMC8157445; doi:10.1186/s40168-021-01067-0)
Supplement: Supplementary file 2 — Additional file 1: Supplementary Figure S1. Benzonase digest approach efficiently depletes dead bacteria and free bacterial DNA reads. Microbial DNA was extracted using a conventional approach (CA) or the benzonase-digest approach (BDA) before bacterial lysis. a) Microbiome analysis of a skin mock community supplemented with live E. coli cells, heat inactivated (hi) E. coli cells or E. coli free DNA. b) Relative abundance reads resulting from E. coli in the mock community. c) Microbiome analysis at genus level of forearm skin samples spiked with hi E. coli or E. coli free DNA. d) Relative abundance of E. coli reads in spiked skin samples. P valued were calculated using Wilcoxon-Mann-Whitney test. * p ≤ 0.05, ** p ≤ 0.01, *** p≤ 0.001. [file 40168_2021_1067_MOESM2_ESM.pdf]

Supplementary Figure S1

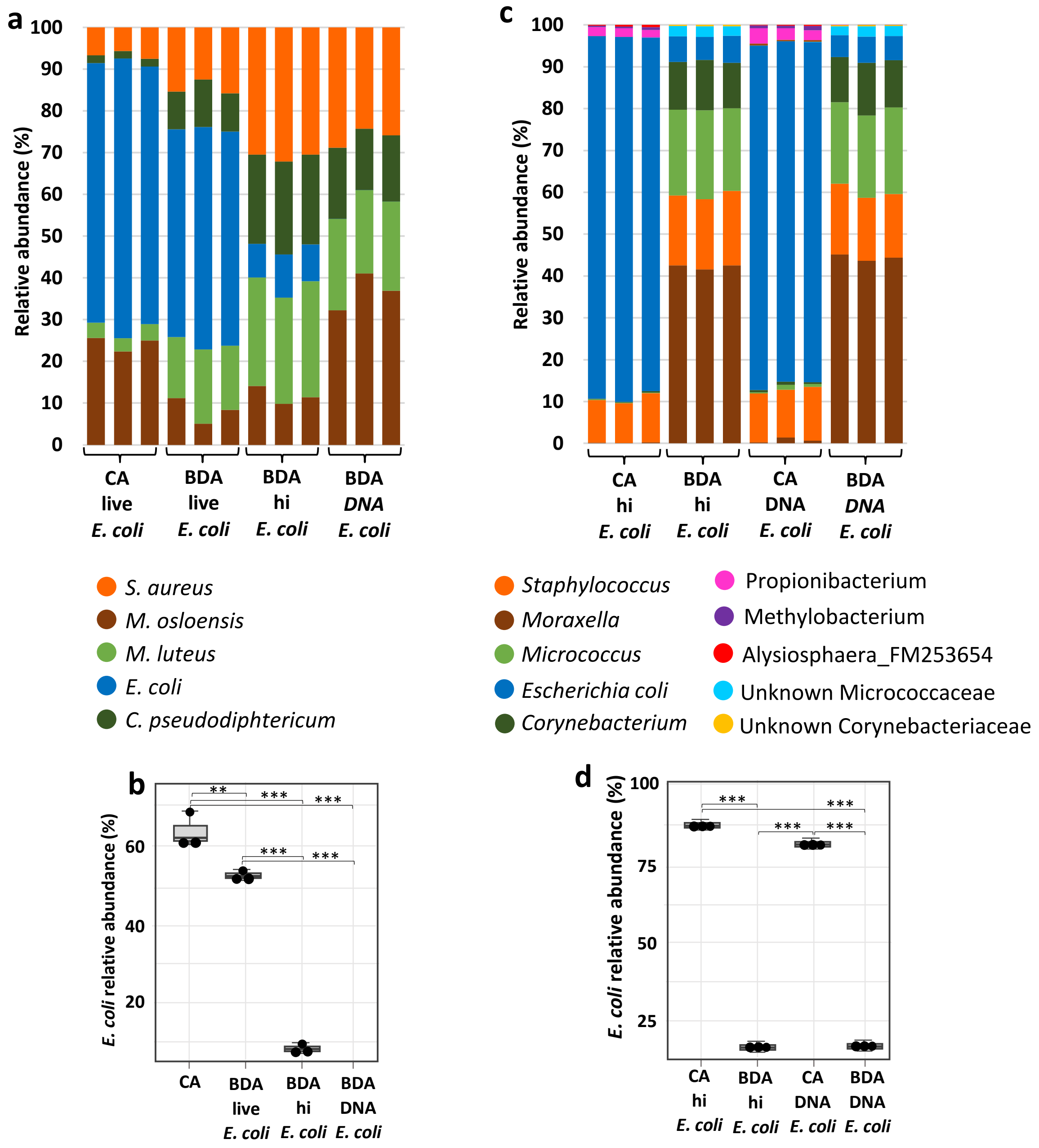

Benzonase digest approach efficiently depletes dead bacteria and free bacterial DNA reads. Microbial DNA was extracted using a conventional approach (CA) or the benzonase-digest approach (BDA) before bacterial lysis. **a)** Microbiome analysis of a skin mock community supplemented with live *E. coli* cells, heat inactivated (hi) *E. coli* cells or *E. coli* free DNA. **b)** Relative abundance reads resulting from *E. coli* in the mock community. **c)** Microbiome analysis at genus level of forearm skin samples spiked with *hi E. coli* or *E. coli* free DNA. **d)** Relative abundance of *E. coli* reads in spiked skin samples. P valued were calculated using Wilcoxon-Mann-Whitney test. * $p \leq 0.05$, ** $p \leq 0.01$, *** $p \leq 0.001$.
