# Supplementary material for: Pre-digest of unprotected DNA by Benzonase improves the representation of living skin bacteria and efficiently depletes host DNA
Source: Microbiome. 2021 May 26;9:123. doi: 10.1186/s40168-021-01067-0 (PMC8157445; doi:10.1186/s40168-021-01067-0)
Supplement: Supplementary file 3 — Additional file 2: Supplementary Figure S2. Benzonase digest approach efficiently depletes dead bacteria and host DNA reads with no impact on microbiota composition. a) DNA yields from a skin mock community supplemented with 3×10^5^ PBMCs/sample and processed with different extraction methods: HA (Human blood kit), CA (conventional approach for bacteria DNA extraction) and the BDA (benzonase digest approach). b) Real-time PCR plot of human DNA reads corresponding to the mock community supplemented with PBMCs extracted using different approaches. c) The principal coordinate analysis plot (PCoA) of β-diversity shows the ability of BDA to eliminate human DNA reads from PBMCs as well as DNA from heat inactivated bacterial cells (from P. aeruginosa and P. mirabilis) and free bacterial DNA (B. simplex). BDA (benzonase-digest approach), NDA (non-benzonase-digest approach). PBMCs (human peripheral blood mono nuclear cells). Live (mock community comprising living bacteria), hi (heat inactivated P. aeruginosa and P. mirabilis), DNA (free DNA from B. simplex). [file 40168_2021_1067_MOESM3_ESM.pdf]

Supplementary Figure S2

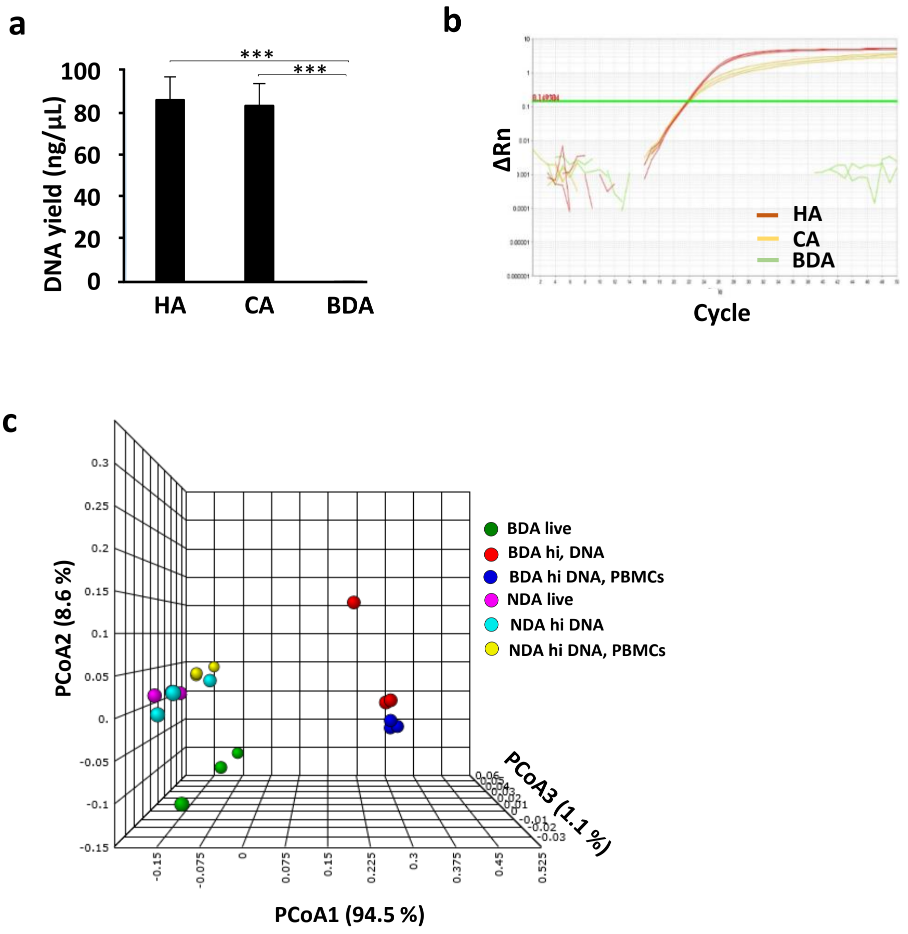

Benzonase digest approach efficiently depletes dead bacteria and host DNA reads with no impact on microbiota composition. a) DNA yields from a skin mock community supplemented with 3×10^5 PBMCs/sample and processed with different extraction methods: HA (Human blood kit), CA (conventional approach for bacteria DNA extraction) and the BDA (benzonase digest approach). **b)** Real-time PCR plot of human DNA reads corresponding to the mock community supplemented with PBMCs extracted using different approaches. **c)** The principal coordinate analysis plot (PCoA) of β-diversity shows the ability of BDA to eliminate human DNA reads from PBMCs as well as DNA from heat inactivated bacterial cells (from *P. aeruginosa* and *P. mirabilis*) and free bacterial DNA (*B. simplex*). BDA (benzonase-digest approach), NDA (non-benzonase-digest approach). PBMCs (human peripheral blood mono nuclear cells). Live (mock community comprising living bacteria), hi (heat inactivated *P. aeruginosa* and *P. mirabilis*), DNA (free DNA from *B. simplex*)
