# Supplementary material for: Pre-digest of unprotected DNA by Benzonase improves the representation of living skin bacteria and efficiently depletes host DNA
Source: Microbiome. 2021 May 26;9:123. doi: 10.1186/s40168-021-01067-0 (PMC8157445; doi:10.1186/s40168-021-01067-0)
Supplement: Supplementary file 4 — Additional file 3: Supplementary Figure S3. Benzonase digest approach efficiently depletes dead bacteria and host DNA from metagenomics reads. a) Metagenomics read counts from skin mock community samples (hi, DNA), supplemented or not with 105 PBMCs and processed either with BDA or NDA. b) Human and bacteria metagenomics read counts in mock community samples (hi, DNA). c) Relative abundance of OTUs detected in metagenomics reads. d) Relative abundance of OTUs obtained from skin mock community supplemented or not with human PBMCs. The taxonomy analysis based on metagenomics data has been performed using MetaPhlAn 3.0 tool. BDA (Benzonase-digest approach), NDA (Non-Benzonase-digest approach). PBMCs (human peripheral blood mono nuclear cells). OTUs (Operational taxonomic units), hi, DNA (Skin mock community with heat inactivated P. aeruginosa and P. mirabilis and free bacterial DNA from B. simplex). [file 40168_2021_1067_MOESM4_ESM.pdf]

Supplementary Figure S3

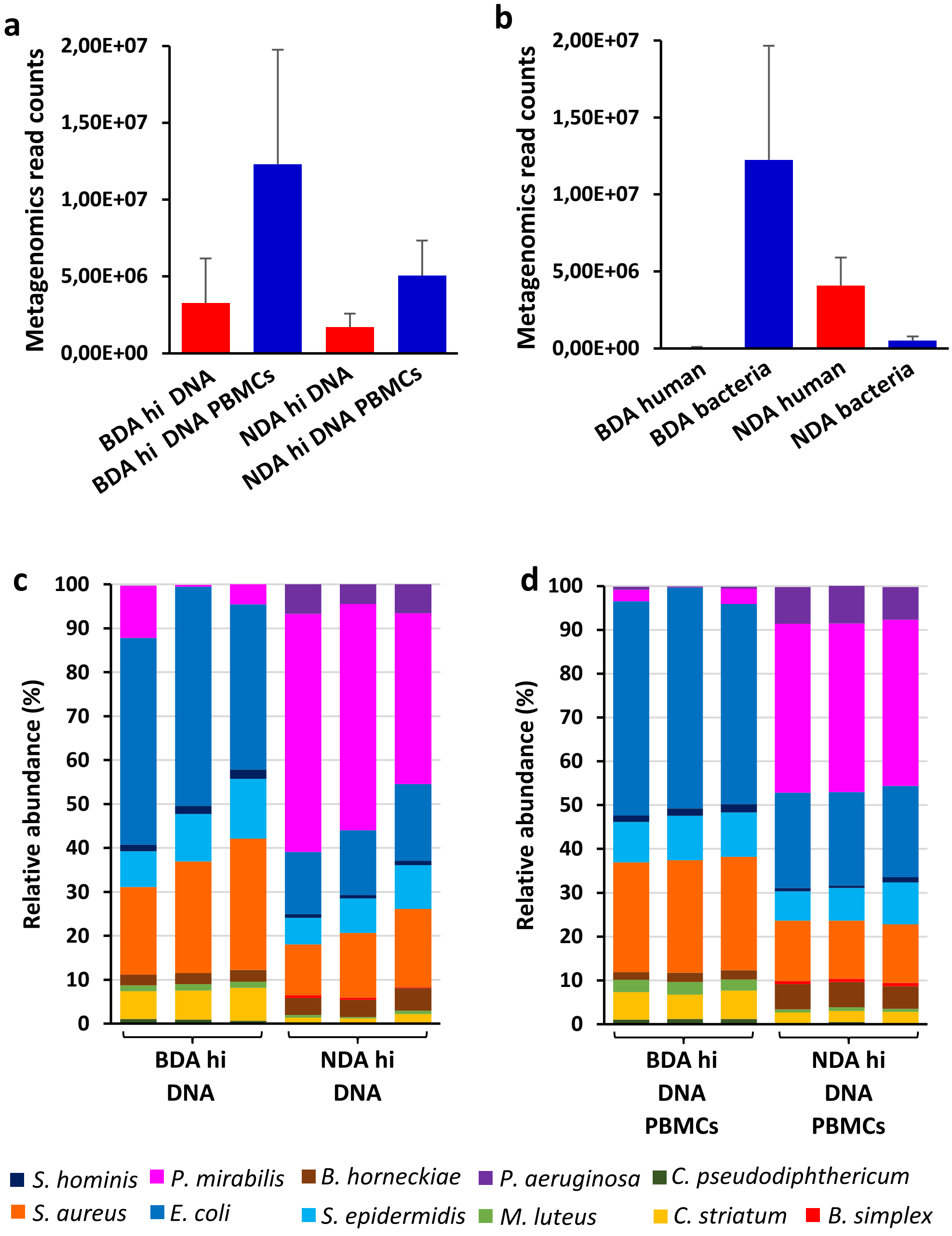

Benzonase digest approach efficiently depletes dead bacteria and host DNA from metagenomics reads. **a)** Metagenomics read counts from skin mock community samples (hi, DNA), supplemented or not with 10^5 PBMCs and processed either with BDA or NDA. **b)** Human and bacteria metagenomics read counts in mock community samples (hi, DNA). **c)** Relative abundance of OTUs detected in metagenomics reads. **d)** Relative abundance of OTUs obtained from skin mock community supplemented or not with human PBMCs. The taxonomy analysis based on metagenomics data has been performed using MetaPhlAn 3.0 tool. BDA (benzonase-digest approach), NDA (non-benzonase-digest approach). PBMCs (human peripheral blood mono nuclear cells). OTUs (Operational taxonomic units), hi, DNA (Skin mock community with heat inactivated *P. aeruginosa* and *P. mirabilis* and free bacterial DNA from *B. simplex*).
