# Supplementary material for: Pre-digest of unprotected DNA by Benzonase improves the representation of living skin bacteria and efficiently depletes host DNA
Source: Microbiome. 2021 May 26;9:123. doi: 10.1186/s40168-021-01067-0 (PMC8157445; doi:10.1186/s40168-021-01067-0)
Supplement: Supplementary file 5 — Additional file 4: Supplementary Figure S4. The Benzonase approach generates less diversity bias compared to CA in low bacterial DNA input samples. The mock community of living bacteria (108 CFU/sample) was diluted to 107, 105 and 103 CFU/sample and then DNA extracted by the CA or the BDA. Taxonomic binning of bacterial taxa in samples processed by a) BDA or b) CA. c, d) Richness e, f) Shannon effective and g, h) β-diversity analysis of the mock community after dilution and extraction using BDA or CA. BDA (Benzonase digest approach), CA (conventional approach). * p ≤ 0.05, ** p ≤ 0.01, *** p ≤ 0.001. [file 40168_2021_1067_MOESM5_ESM.pdf]

Supplementary Figure 4

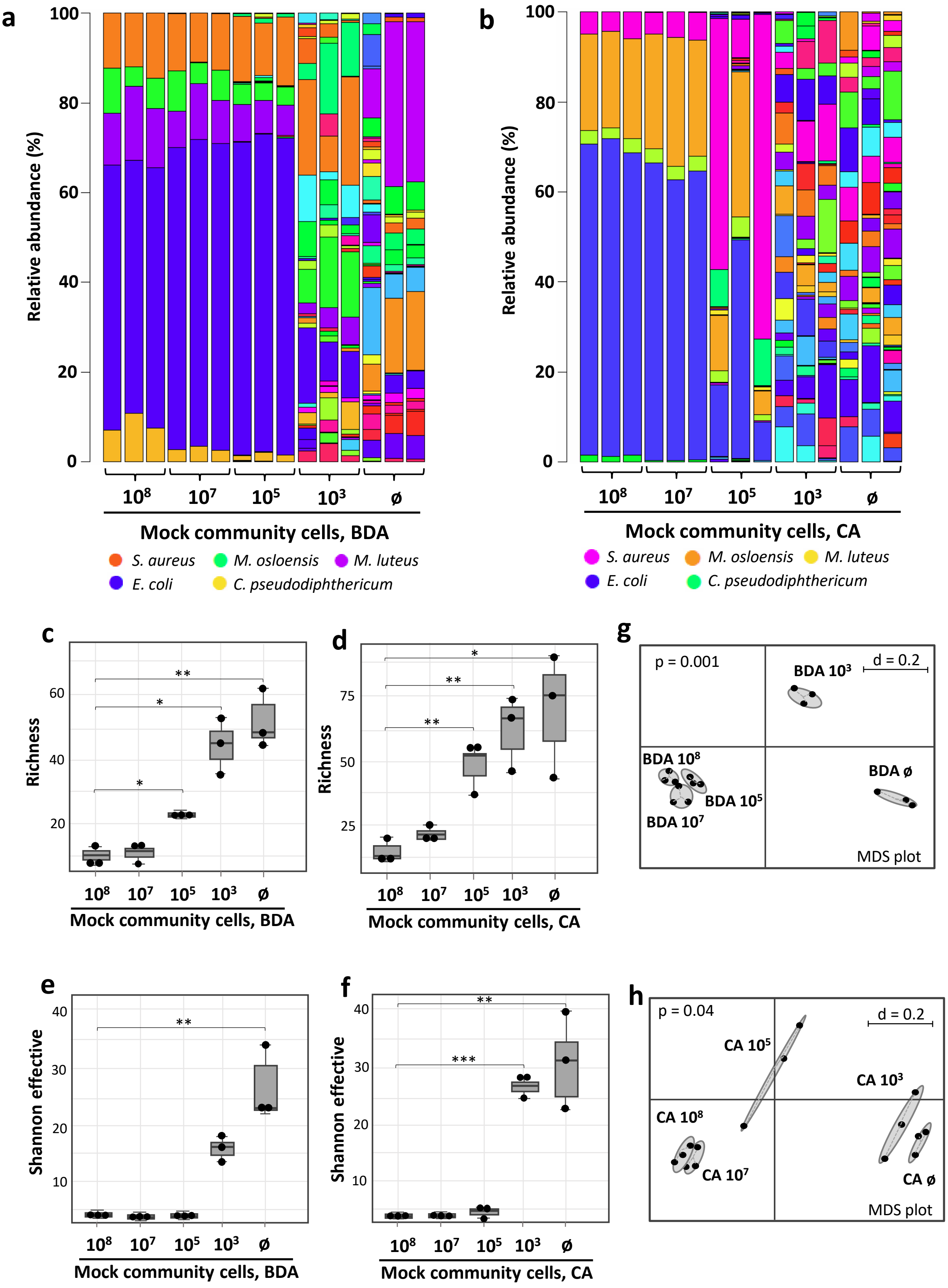

The benzonase approach generates less diversity bias compared to CA in low bacterial DNA input samples. The mock community of living bacteria (10^8 CFU/sample) was diluted to 10^7, 10^5 and 10^3 CFU/sample and then DNA extracted by the CA or the BDA. Taxonomic binning of bacterial taxa in samples processed by **a)** BDA or **b)** CA. **c, d)** Richness **e, f)** Shannon effective and **g, h)** β-diversity analysis of the mock community after dilution and extraction using BDA or CA. BDA (Benzonase digest approach), CA (conventional approach). * $p \leq 0.05$, ** $p \leq 0.01$, *** $p \leq 0.001$.
