# Supplementary material for: Pre-digest of unprotected DNA by Benzonase improves the representation of living skin bacteria and efficiently depletes host DNA
Source: Microbiome. 2021 May 26;9:123. doi: 10.1186/s40168-021-01067-0 (PMC8157445; doi:10.1186/s40168-021-01067-0)
Supplement: Supplementary file 6 — Additional file 5: Supplementary table 01. Abundant OTUs in the Benzonase digest approach (BDA) upon dilution (OTUs used to constitute the mock community in bold letters). Supplementary table 02. Abundant OTUs in the Non-Benzonase digest approach (NDA) upon dilution (OTUs used to constitute the mock community in bold letters). Supplementary table 03. Abundant OTUs (> 1%) in the benzonase digest approach (BDA) upon dilution (small mock community, OTUs used to constitute the mock community in bold letters). Supplementary table 04. Abundant OTUs (> 1%) in the conventional approach (CA) upon dilution (small mock community, OTUs used to constitute the mock community in bold letters). [file 40168_2021_1067_MOESM6_ESM.pdf]

Supplementary table 01: Abundant OTUs in the Benzonase digest approach (BDA) upon dilution (OTUs used to constitute the mock community in bold letters)

	**BDA_10^{8}**	**BDA_10^{7}**	**BDA_10^{5}**	**BDA_10^{3}**	**Control**
E. coli	18.94	18.82	25.03	13.17	0.01
S. aureus	23.09	23.57	17.11	17.35	14.53
P. mirabilis	17.78	16.34	16.38	9.74	0.30
P. aeruginosa	17.03	16.27	15.91	8.79	0.13
S. epidermidis	4.44	7.29	6.19	10.13	16.73
B. horneckiae	6.42	5.16	4.16	0.55	0.00
C. striatum	3.78	5.40	2.57	2.45	2.33
M. luteus	6.19	4.82	2.14	0.88	0.42
C. pseudodiphtheriticum	1.44	1.53	0.73	0.29	0.00
S. hominis	0.88	0.74	0.14	0.11	0.09
C. acnes	0.00	0.00	0.15	2.34	6.89
A. nanhaiticus	0.00	0.00	0.02	1.56	5.12
P. panacisoli	0.00	0.00	0.02	0.19	1.07
M. mitochondrii	0.00	0.00	7.70	8.09	8.05
U. oligocarboniphilum	0.00	0.00	0.03	1.53	5.59
C. bouchesdurhonense	0.00	0.00	0.11	2.07	2.42
C. coyleae	0.00	0.00	0.04	1.66	1.75
S. aerolata	0.00	0.00	0.26	0.79	0.63
Staphylococcus sp	0.02	0.04	0.05	0.20	0.69
K. tytonicola	0.00	0.00	0.06	0.82	2.60
A. muciniphila	0.00	0.00	0.00	0.00	0.68
G. thermoleovorans	0.00	0.00	0.00	0.71	0.00
S. mitis	0.00	0.00	0.02	1.32	1.67
S. dysgalactiae	0.00	0.00	0.07	1.03	1.01
A. proteolyticus	0.00	0.00	0.01	0.46	0.55
L. taiwanensis	0.00	0.00	0.01	0.41	1.01
S. thermophilus	0.00	0.00	0.02	0.17	0.58
L. reuteri	0.00	0.00	0.02	0.20	1.17
Muribaculaceae PAC00679	0.00	0.00	0.00	0.53	0.00
C. gracilis	0.00	0.00	0.00	0.11	0.90
Chroococcidiopsis sp	0.00	0.00	0.01	0.31	3.00
S.anginosus	0.00	0.00	0.00	0.11	1.65
G. leopoldii	0.00	0.00	0.00	0.00	0.51
C. haliotis	0.00	0.00	0.01	0.00	2.02
Saccharimonas sp	0.00	0.00	0.00	0.00	0.62
D. muris	0.00	0.00	0.00	0.00	0.61
Atopostipes sp	0.00	0.00	0.01	0.00	1.20
B. intermedia	0.00	0.00	0.00	0.00	0.55
Hymenobacter sp	0.00	0.00	0.00	0.00	0.56
M. curtisii	0.00	0.00	0.00	0.05	0.51

Supplementary table 02: Abundant OTUs in the Non-Benzonase digest approach (NDA) upon dilution (OTUs used to constitute the mock community in bold letters)

	NDA_108	NDA_107	NDA_105	NDA_103	Control
E. coli	17.19	9.41	6.99	6.20	0.11
S. aureus	18.40	18.32	19.06	19.42	12.72
P. mirabilis	17.10	23.40	21.57	7.45	8.13
P. aeruginosa	15.98	16.64	16.07	5.52	0.28
S. epidermidis	3.64	5.12	7.75	11.16	26.22
B. horneckiae	16.50	16.54	11.47	5.00	0.02
C. striatum	2.37	3.08	2.22	2.94	2.11
M. luteus	5.97	5.08	1.94	1.91	0.54
C. pseudodiphtheriticum	0.95	0.59	0.48	0.00	0.00
S. hominis	1.88	1.78	0.45	0.19	0.15
C. acnes	0.00	0.00	0.12	1.52	2.07
A. nanhaiticus	0.00	0.00	0.05	1.42	0.91
P. panacisoli	0.00	0.00	0.02	0.59	0.54
M. mitochondrii	0.00	0.00	0.24	7.61	9.20
U. oligocarboniphilum	0.00	0.00	0.04	3.64	5.08
C. bouchesdurhonense	0.00	0.00	5.16	3.43	2.96
C. coyleae	0.00	0.00	0.31	2.04	0.74
S. aerolata	0.00	0.00	0.01	0.54	0.19
Staphylococcus sp	0.01	0.02	0.10	0.45	0.97
K. tytonicola	0.00	0.00	0.01	0.45	1.73
A. muciniphila	0.00	0.00	0.00	0.39	0.33
S. mitis	0.00	0.00	0.01	0.67	0.48
S. dysgalactiae	0.00	0.01	0.08	1.59	2.20
L. taiwanensis	0.00	0.00	0.01	1.97	1.37
C. appendicis	0.00	0.00	0.69	0.15	0.47
S. thermophilus	0.00	0.00	0.01	0.97	0.12
C. thomssenii	0.00	0.00	0.68	0.18	0.33
L. reuteri	0.00	0.00	0.01	0.85	1.06
M. durans	0.00	0.00	0.00	0.00	0.52
M.atlantae	0.00	0.00	1.07	0.40	0.19
C. gracilis	0.00	0.00	0.00	0.08	0.87
Chroococcidiopsis sp	0.00	0.00	0.01	0.65	0.18
A. DQ811848	0.00	0.00	0.00	0.00	1.70
M.atlantae	0.00	0.00	0.77	0.19	0.08
B. coagulans	0.00	0.00	0.00	0.19	0.78
Muribaculum RAYW	0.00	0.00	0.00	0.00	0.56
D. muris	0.00	0.00	0.00	0.00	0.56
L. PAC000671	0.00	0.00	0.00	0.00	0.53
Muribaculaceae PAC01127	0.00	0.00	0.00	0.00	0.53
A. lwoffii	0.00	0.00	0.00	0.00	0.50

Supplementary table 03: Abundant OTUs (> 1%) in the benzonase digest approach (BDA) upon dilution (small mock community, OTUs used to constitute the mock community in bold letters)

	**BDA_10^8**	**BDA_10^7**	**BDA_10^5**	**BDA_10^3**	**Control**
E. coli	57.88	68.13	70.46	11.96	2.67
S. aureus	12.86	12.13	13.85	17.84	0.08
M. luteus	13.73	10.06	7.48	4.35	0.30
M. osloensis	7.01	6.78	4.21	12.62	0.18
C. pseudodiphthericum	8.42	2.82	1.45	3.30	0.00
S. thermophilus	0.00	0.00	0.80	10.44	0.00
S. epidermidis	0.01	0.00	0.06	2.22	0.08
O. anthropi	0.00	0.00	0.06	1.78	0.00
Lawsonella sp	0.00	0.00	0.25	0.74	1.05
R. erythropolis	0.00	0.00	0.32	5.08	2.14
S. hominis	0.05	0.04	0.13	2.67	0.38
A. senegalensis	0.00	0.00	0.01	2.55	0.49
S. epidermidis	0.03	0.01	0.05	1.39	0.18
D. acidovorans	0.00	0.00	0.16	1.18	1.78
B. elkanii	0.00	0.00	0.04	0.92	2.41
U. oligocarboniphilum	0.00	0.00	0.13	2.95	1.77
R. solanacearum	0.00	0.00	0.02	1.22	0.00
C. genitalium	0.00	0.00	0.00	1.70	0.05
M. populi	0.00	0.00	0.00	0.00	6.99
PAC000328 EU861940	0.00	0.00	0.00	0.00	1.77
L. reuteri	0.00	0.00	0.00	0.00	1.30
PAC000016 JQ427566	0.00	0.00	0.00	0.00	1.32
EU289441 JF168546	0.00	0.00	0.00	0.00	1.11
PAC001066 PAC001066	0.00	0.00	0.00	0.00	1.12
L. gasseri	0.00	0.00	0.00	0.00	12.07
C. PAC001294	0.00	0.00	0.00	0.00	1.39
Chthoniobacteraceae EF018472	0.00	0.00	0.00	0.00	1.27
M. radiotolerans	0.00	0.00	0.00	0.00	1.66
Muribaculaceae. PAC001070	0.00	0.00	0.00	0.00	3.97
S. gilvigriseus	0.00	0.00	0.00	0.00	2.38
Muribaculaceae. PAC001112	0.00	0.00	0.00	0.00	5.49
Alistipes PAC002444	0.00	0.00	0.00	0.00	3.15
S. maltophilia	0.00	0.00	0.01	1.70	0.00
Lachnospiraceae KE159571	0.00	0.00	0.00	0.00	4.18
Muribaculaceae PAC001075	0.00	0.00	0.00	0.00	5.07
Muribaculaceae PAC001064	0.00	0.00	0.00	0.00	2.39
A. towneri	0.00	0.00	0.00	0.00	1.87
Muribaculum sp	0.00	0.00	0.00	0.00	2.07
A.radioresistens	0.00	0.00	0.00	0.00	1.70
Muribaculaceae. PAC001112	0.00	0.00	0.00	0.00	2.32
Muribaculaceae. PAC002480	0.00	0.00	0.00	0.00	2.37

Supplementary table 04: Abundant OTUs (> 1%) in the conventional approach (CA) upon dilution (small mock community, OTUs used to constitute the mock community in bold letters)

	CA_10^8	CA_10^7	CA_10^5	CA_10^3	Control
E. coli	69.03	64.29	24.32	5.82	0.01
S. aureus	5.04	5.57	45.52	8.44	6.29
M. luteus	2.82	3.20	2.83	0.03	0.00
M. osloensis	21.72	26.63	16.58	4.34	2.37
C. pseudodiphthericum	1.37	0.29	0.20	0.00	0.00
A. xylosoxidans	0.00	0.00	0.00	3.31	1.65
S. soli	0.00	0.00	0.00	2.13	0.00
B. robiniae	0.00	0.00	0.00	1.91	0.00
D. acidovorans	0.00	0.00	0.04	1.69	0.00
U. oligocarboniphilum	0.00	0.00	0.04	2.94	6.28
R.pickettii	0.00	0.00	0.28	2.52	5.07
M. mucogenicum	0.00	0.00	0.10	1.87	2.92
H. marinus	0.00	0.00	0.00	1.59	0.09
B. simplex	0.00	0.00	0.00	1.35	0.00
R. nasimurium	0.00	0.00	0.10	2.60	0.44
A. johnsonii	0.00	0.00	0.01	3.96	4.86
A.GQ051146	0.00	0.00	0.00	2.91	0.00
R. erythropolis	0.00	0.01	0.25	5.71	1.99
S. maltophilia	0.00	0.00	0.02	0.46	2.26
B. pseudomycoides	0.00	0.00	0.11	4.37	7.40
S. gilvigriseus	0.00	0.00	0.49	5.42	4.46
Lawsonella sp	0.00	0.00	0.06	1.48	0.33
S.arlettae	0.00	0.00	0.01	1.51	0.15
Lawsonella sp	0.00	0.00	0.11	0.98	1.83
M. luteus	0.00	0.00	0.02	4.06	0.33
P. putida	0.00	0.00	0.01	1.78	0.59
K. flava	0.00	0.00	0.00	1.06	0.00
R. solanacearum	0.00	0.00	0.06	1.49	0.78
C. aurimucosum	0.00	0.00	0.01	1.11	0.43
C. mycetoides	0.00	0.00	0.01	1.14	0.00
A. viridans	0.00	0.00	0.01	0.00	1.05
D. incerta	0.00	0.00	0.00	0.00	1.13
D. polyhydroxybutyrativorans	0.00	0.00	0.02	0.00	3.06
S. nitritireducens	0.00	0.00	0.00	0.00	2.16
S. hankookensis	0.00	0.00	0.16	0.00	4.09
S. sacchari	0.00	0.00	0.00	0.00	2.22
Acetobacteraceae. AJ292598	0.00	0.00	0.00	0.00	1.45
S. aquatica	0.00	0.00	0.03	0.00	2.14
V. thermophilum	0.00	0.00	0.07	0.00	1.84
S. multivorans	0.00	0.00	0.00	0.00	1.14
T. thermophilus	0.00	0.00	0.00	0.00	1.04
N. canariense	0.00	0.00	0.00	0.00	1.45
