# Supplementary material for: Pre-digest of unprotected DNA by Benzonase improves the representation of living skin bacteria and efficiently depletes host DNA
Source: Microbiome. 2021 May 26;9:123. doi: 10.1186/s40168-021-01067-0 (PMC8157445; doi:10.1186/s40168-021-01067-0)
Supplement: Supplementary file 7 — Additional file 6: Table 01. 16S rRNA gene amplicon reads per sample after every processing step. Table 02. Metagenomics reads per sample after every processing step. Table 03. Metagenomics based taxa relative abundances. Table 04. Metagenomics based taxa relative abundances in sampled containing PBMCs. [file 40168_2021_1067_MOESM7_ESM.pdf]

Table 01: 16S amplicon reads per sample after every processing step.

Sample_ID	Demultiplexing	Merging	EE-filtering	Chimeras-Artifacts	Abundance filter
BDA Live 1	64376	46687	46634	30716	30443
BDA Live 2	63579	46281	46218	30113	29844
BDA Live 3	57014	42030	41975	27392	27150
NDALive 1	72804	56017	55944	36934	36649
NDALive 2	68344	52509	52451	34763	34517
NDALive 3	63472	49072	49021	32174	31906
BDA hi 1	55485	40808	40738	29456	29290
BDA hi 2	52441	38148	38084	27140	26990
BDA hi 3	56359	39652	39591	28021	27887
NDA hi 1	96259	74127	74057	48938	48529
NDA hi 2	61878	48036	47978	31537	31277
NDA hi 3	74659	55777	55707	35424	35074
BDA hi DNA 1	58365	43084	43016	33607	33431
BDA hi DNA 2	56786	41644	41567	30612	30446
BDA hi DNA 3	64375	46433	46349	33894	33686
NDAhi DNA 1	48276	37684	37643	25205	25024
NDAhi DNA 2	80056	60811	60744	39656	39337
NDAhi DNA 3	73894	55466	55397	36179	35902

Table 02: Metagenomics reads per sample after every processing step.

Sample_ID	DNA ng/µl	Raw Reads	Adapter removal	Quality filtering	human reads	non-human reads
BDA 1	0.61	8445993	8147200	7282731	3607	7279124
BDA 2	1.98	2168592	2144669	2032292	316	2031976
BDA3	2.5	480960	474169	444292	2635	441639
BDA control	0	810	666	536	145	391
NDA 1	5.4	3257170	3164944	2837747	741	2837006
NDA 2	6.9	1864545	1796326	1590207	1393	1588814
NDA 3	6.8	719178	693425	670545	725	669793
NDA control	0	11374	5749	4345	1903	2442
BDA PBMC 1	2.54	5908301	5838956	5470908	23015	5447893
BDA PBMC 2	2.56	9703454	9519374	8787552	22296	8765256
BDA PBMC 3	2.72	24951792	24489770	22659487	100043	22559444
BDA control	0	810	666	536	145	391
NDA PBMC 1	22.2	4423878	4338383	4251904	3419946	831958
NDA PBMC 2	27.2	9494653	9237603	8150548	6571662	156886
NDA PBMC 3	29.2	3266623	3170090	2726353	2224846	501507
NDA control	0	11374	5749	4345	1903	2442

Table 03: Metagenomics based taxa relative abundances

	BDA1	BDA2	BDA3	NDA1	NDA2	NDA3
C. pseudodiphtheriticum	1.11	0.92	0.66	0.20	0.19	0.09
C. striatum	6.27	6.63	7.50	1.17	1.04	2.10
M. luteus	1.33	1.44	1.45	0.66	0.28	0.82
B. horneckiae	2.46	2.55	2.61	3.86	3.93	5.08
B. simplex	0.02	0.00	0.00	0.57	0.48	0.19
S. aureus	19.96	25.40	29.91	11.63	14.72	17.85
S. epidermidis	8.12	10.78	13.60	6.00	7.87	9.98
S. hominis	1.46	1.84	2.14	0.87	0.78	1.00
E. coli	47.09	49.88	37.64	14.19	14.71	17.48
P. mirabilis	11.87	0.43	4.50	54.15	51.57	38.85
P. aeruginosa	0.01	0.00	0.00	7.22	4.97	7.14

Table 04: Metagenomics based taxa relative abundances in sampled containing PBMCs

	BDA1-PBMCs	BDA2-PBMCs	BDA3-PBMCs	NDA1-PBMCs	NDA2-PBMCs	NDA3-PBMCs
C. pseudodiphtheriticum	1.04	1.21	1.18	0.30	0.50	0.24
C. striatum	6.31	5.54	6.49	2.37	2.56	2.55
M. luteus	2.80	2.91	2.56	0.75	0.81	0.73
B. horneckiae	1.75	2.07	2.10	5.72	5.76	5.14
B. simplex	0.00	0.00	0.00	0.69	0.73	0.76
S. aureus	25.01	25.72	25.87	13.84	13.26	13.39
S. epidermidis	9.24	10.08	10.15	6.70	7.46	9.56
S. hominis	1.48	1.71	1.86	0.73	0.62	1.22
E. coli	48.86	50.38	45.69	21.72	21.30	20.79
P. mirabilis	2.61	0.05	3.50	38.56	38.46	37.97
P. aeruginosa	0.70	0.24	0.41	8.41	8.57	7.43
